# DiffInt: A Diffusion Model for Structure-Based Drug Design with Explicit Hydrogen Bond Interaction Guidance

**DOI:** 10.1021/acs.jcim.4c01385

**Published:** 2024-12-19

**Authors:** Masami Sako, Nobuaki Yasuo, Masakazu Sekijima

**Affiliations:** † Department of Computer Science, Institute of Science Tokyo, Yokohama, Kanagawa 226-8501, Japan; ‡ Academy for Convergence of Materials and Informatics (TAC-MI), Institute of Science Tokyo, Meguro-ku, Tokyo 152-8550, Japan

## Abstract

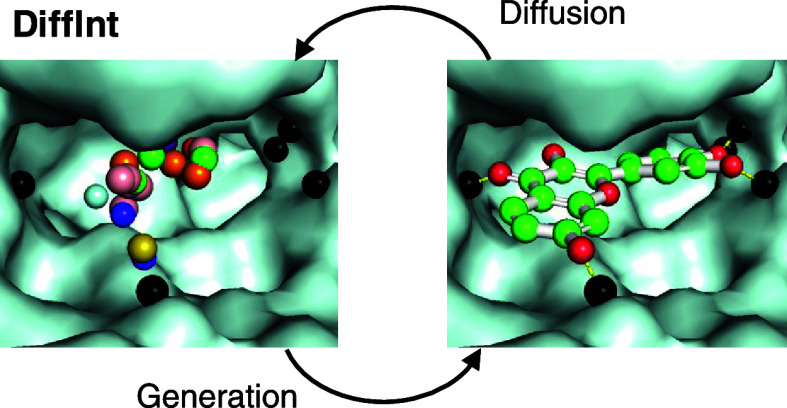

The design of drug molecules is a critical stage in the drug discovery process. The structure-based drug design has long played an important role in efficient development. Significant progress has been made in recent years in the generation of 3D molecules via deep generation models. However, while many existing models have succeeded in incorporating structural information on target proteins, they have not been able to address important interactions between protein and drug molecules, especially hydrogen bonds. In this study, we propose DiffInt as a novel structure-based approach that explicitly addresses interactions. The model naturally incorporates hydrogen bonds between protein and ligand molecules by treating them as pseudoparticles. The experimental results show that DiffInt reproduces hydrogen bonds, and the hydrogen binding energies significantly outperform those of existing models. To facilitate the use of our tool for generating new drug molecules based on any protein's three-dimensional structure, we have made the source code and trained model available on GitHub (https://github.com/sekijima-lab/DiffInt) under the MIT license, with the execution environment provided on Google Colab.

## Introduction

The primary goal of drug discovery is to identify novel molecules that bind to specific pockets of target proteins and possess desirable pharmacological properties. These drug-like molecules can then inhibit or activate specific biological functions. Structure-based drug design (SBDD) began to attract attention in the 1980s to design ligand molecules effectively based on experimental knowledge of the three-dimensional structure of proteins.^1−4^ It is useful information to have similar 3D structures and interactions for designing ligand molecules, as they are based on essential protein–ligand binding interactions such as hydrogen bonds, hydrophobic interactions, aromatic interactions, ionic interactions, and van der Waals forces, and so on.^5−7^ Among these interactions, research focusing particularly on hydrogen bonding and hydrophobic interactions has been conducted.^8,9^ When analyzing 19,443 protein–ligand pair data in PDBbind v2020,^10^ 91.4% of the data contain hydrogen bonds with an average of 5.5 hydrogen bonds per molecule, indicating that hydrogen bonds play an important role in the binding between protein and ligand molecules in many cases.

Traditionally, SBDD has been carried out via high-throughput experimental screening^11,12^ or virtual screening^13,14^ via large chemical databases.^15−20^ With advancements in structural biology, such as X-ray crystallography^21,22^ and cryo-electron microscopy,^23,24^ the number of crystal structures registered in the Protein Data Bank (PDB)^15^ has exceeded 220,000. The development of innovative deep learning methods in recent years has led to remarkable improvements in protein structure prediction accuracy,^25−27^ opening new opportunities for SBDD applications.

The chemical space is estimated to contain approximately 10^60^ synthetically accessible molecules, with the latest research focusing on drug-like molecules estimating that GDB-17 includes compounds on the order of 10^11^.^28^ Virtual screening, such as docking simulations, on these databases requires enormous computational costs for exhaustive exploration. Furthermore, virtual screening via these databases is limited to the knowledge of previously studied regions, making it difficult to explore unknown molecular structures that are not in the databases.

In recent years, the remarkable development of deep learning methods has facilitated efficient exploration of the vast chemical space.^29^ Numerous efforts have been made in ligand-based drug design (LBDD). The methods for handling molecules can be classified into three types: one-dimensional strings (e.g., SMILES^30^ and SELFIES^31^), two-dimensional molecular graphs, and three-dimensional molecular graphs. Most models, however, adopt a 1D or 2D molecular representation as a simpler method. Various deep learning models such as variational autoencoders (VAEs),^32−34^ generative adversarial networks (GANs),^35,36^ normalizing flows,^37,38^ recurrent neural networks (RNNs),^39−41^ transformers,^42^ autoregressive models,^43,44^ and so on have been successfully used in the field of molecule generation, depending on the types of data and objectives.

In contrast to the simplicity of dealing with 1D or 2D data, the inability to represent the intrinsic 3D information on the molecules is a problem. In recent years, several models that handle 3D graphs have appeared,^45,46^ and drug discovery research is accelerating. These LBDD methods can learn the distribution of chemical structures from large compound databases and generate new structures, but they are inefficient in generating molecules that exhibit high binding affinity, because they have no pocket information on the target protein expected to bind.

In deep learning with SBDD, there are two ways to incorporate protein information: one is to use the docking score as an evaluation function, and the other is to use the 3D structure directly as a condition. In the first case, docking evaluation is performed on the target protein and the ligand molecule generated via the same framework as the LBDD method, and this evaluation score is used as a reward function to bias ligand generation.^47,48^

In the latter case, several efforts can be mentioned as relatively simple methods: Xu et al.^49^ generates molecules via a conditional RNN (cRNN) with a features vector of protein pocket, Skalic et al.^50^ generates molecules via VAE from the shape of ligand molecules docked to a protein pocket, and RELATION^51^ learns geometric features of protein–ligand complexes and employs a pharmacophore-based conditional VAE for molecule generation. While these studies consider the 3D information on the protein pocket as input conditions, generated molecules are represented by 1D information (SMILES). It is therefore necessary to generate the 3D structure and search for binding poses for the pocket during postprocessing, and the generated molecules do not sufficiently reflect the 3D information on the protein pocket.

LiGAN^52^ is an early attempt to generate ligand molecules directly in the protein pocket. The method predicts atomic density maps of molecules, requiring subsequent prediction from density maps to molecular structures. In subsequent studies, Pocket2Mol^53^ uses an autoregressive model to directly generate molecules instead of atomic density maps. FLAG^54^ uses a similar autoregressive model to generate molecules by sequentially generating fragments with the aim of generating more realistic molecules. DeepLigBuilder^55^ also applies the autoregressive model to 3D molecule generation by combining it with a Monte Carlo tree search. All of these studies use sequential generation methods, in which errors at each step are carried over to the next step so that errors accumulate as the generation process proceeds. As a result, the autoregressive model in which atoms are sequentially generated is more likely to produce unrealistic molecules and tends to bias toward certain structures because the structure selected early in the generation process has a large impact on subsequent sampling.^56^

To overcome these limitations of sequential generation, diffusion models,^57,58^ which have recently attracted significant attention in the field of image generation, have been applied to the field of molecule generation.^56,59−61^ In the diffusion model, molecules are represented as point clouds in 3D space via E(3)-equivariant graph neural networks (EGNNs),^56^ and all the atoms are generated in parallel, so there is no bias as described above. DiffSBDD^59^ and TargetDiff^62^ are both diffusion-based molecular generation models that directly generate molecules within protein binding pockets. These models achieve molecular generation as SBDD by targeting only the ligand molecules in the forward and reverse diffusion processes, while treating the protein structure as a fixed conditional input throughout. PMDM^60^ further incorporates varying bond strengths into the model by considering the distances between nodes. DiffLinker^63^ employs a diffusion model to perform linker design to connect between multiple molecular fragments, conditioned on the protein pocket structure. DiffDec^61^ proposes a diffusion based model for generating molecules that performs scaffold decoration subject to protein pockets and molecular scaffolds.

However, current molecular generation models have a limitation in SBDD. They do not sufficiently consider the specific interactions between protein and ligand molecules. This is because structural information on protein binding sites alone is insufficient to capture these crucial interactions.

In this study, we propose a new method, DiffInt, to reconstruct the interaction between protein and ligand molecules in the SBDD via E(3)-EGNNs and a diffusion model. In particular, we have successfully incorporated hydrogen bonds between protein and ligand molecules, which are considered important for binding affinity,^64−67^ explicitly into the model by introducing *interaction particles*, which are pseudoparticles. The *interaction particles* provide information on whether the bond is electron-withdrawing or electron-donating for the ligand molecule and provide information on the distance and direction from a specific protein atom. The introduction of these particles, then, enables the reconstruction of hydrogen bonds with specific protein moieties. By generating molecules with protein pocket and *interaction particles*, we show that our method outperforms the baseline method in terms of reconstructing hydrogen bonds between protein and ligand molecules and, consequently, hydrogen bond energies, which are important for binding affinity, while maintaining the performance of the conventional metrics compared to baseline models.

## Method

### Overview of DiffInt

**Figure 1:**
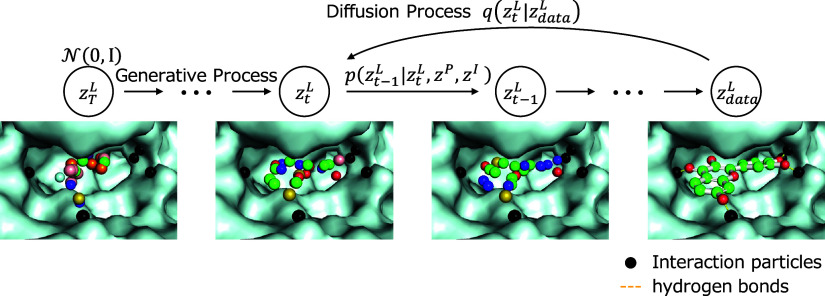
Overview of DiffInt. The diffusion process adds noise to the data to obtain a latent noise representation, resulting in a Gaussian distribution at time step *T* in the center-of-gravity system of the input data. The generative process reconstructs the original data by gradually denoising the noise, and consequently also reconstructs the hydrogen bonds with the protein pocket. In both processes, the protein molecules and hydrogen bonding particles are treated as conditions and remain unchanged.

DiffInt has the ability to generate diverse ligand molecules based on the basis of SBDD considering the interaction, specifically hydrogen bonds between protein pockets and ligand molecules (Figure 1). The hydrogen bonds defined between the acceptor and donor atoms are represented by pseudoparticles called *interaction particles* between those two atoms. In addition, proteins are represented by a graph of the *C*_α_ level. Given protein–ligand pair data, the protein and ligand graphs are represented as  and , respectively. *x*_*i*_ represents the 3D Cartesian coordinate vector, and *h*_*i*_ represents a one-hot vector where protein denotes the residue type and ligand denotes the atom type. The *interaction particles* can be similarly defined as , where  is the same as above and  represnts either the acceptor or donor. Following the framework of the denoising diffusion probabilistic model (DDPM),^58^ the ligand molecules are generated under the conditions of a protein pocket and *interaction particles*. The diffusion model consists of two Markov chains: a diffusion process and a denoising process. The diffusion process adds noise to the data in each step, whereas the denoising process gradually removes noise to reproduce the true data by learning a neural network.

The two-dimensional distribution of protein and ligand particle counts is calculated from the protein–ligand pair data in the training set. The distribution is then smoothed with a Gaussian filter. During the generation process, the number of ligand molecules is determined by sampling from the distribution, conditioned on the number of pocket particles.

### Diffusion Process

The diffusion process involves gradually adding Gaussian noise to the data, creating a latent noise representation from time step *t* = 1··· *T*. The latent noise state *z*_*t*_ at time step t is formulated as these fixed noise processes
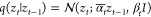
1where α̅_*t*_ controls how much of the original data are preserved, and β_*t*_ controls the magnitude of the noise variance to be added. β_*t*_, known as the noise schedule, follows the relationship 0 < β_0_ < β_1_ <··· < β_*T*_ < 1 and has the relation of  as a variance preserving process.^58^ The entire noising process is formulated as a Markov chain
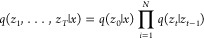
2The noise representation at an arbitrary step t can be expressed in closed form by utilizing the reparameterization trick
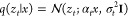
3where  and is scheduled to smoothly transit from α_0_ ≈ 1 to α_*T*_ ≈ 0. The variance preserving process is adopted like eq 1, and thus . Following Kingma et al.,^68^ the signal-to-noise ratio  is introduced to simplify the notation. The denoising process is also a Markov chain, and the *true denoising* transition from time step *t* to *s* < *t* can be expressed in a closed form by adding the original data x as a condition
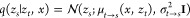
4where the mean μ_*t*→*s*_ and the variance  are defined as follows
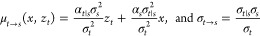
5with  and  following the notation of Hoogeboom et al.^56^

### Generative Process

The generative process aims to reconstruct the original data *x* by sequentially removing noise from the noisy state *z*_*T*_, step by step. eq 4 is not directly applicable when generating new samples because the data *x* is unknown. Therefore, it is replaced with *x̂* predicted by the neural network. The denoising transition distribution *p*_θ_(*z*_*s*_|*z*_*t*_) takes the same form as *q*(*z*_*s*_|*z*_*t*_, *x*); thus, it can be expressed as follows with the predicted variable *x̂*:
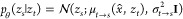
6Ho et al.^58^ discovered that it is easier for a neural network to predict the Gaussian noise ϵ̂_θ_ = ϕ_θ_(*z*_*t*_, *t*) to be removed rather than directly predicting *x̂*. Specifically, by rewriting eq 3 as *z*_*t*_ = α_*t*_*x* + σ_*t*_ϵ with  and replacing ϵ with ϵ̂, then *x̂* can be obtained as follows
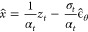
7

### Variational Lower Bound

The neural network is trained to maximize the likelihood of the observed data by optimizing the variational lower bound (VLB)^56,68^ on the data
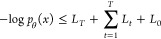
8where *L*_*T*_ = *D*_KL_(*q*(*z*_*T*_|*x*) ∥ *q*(*z*_*T*_)) is the prior loss. In the diffusion model, the prior distribution *p*(*z*_*T*_) is assumed to be a standard normal distribution, and the KL-divergence is used to evaluate the discrepancy between the final latent representation *p*(*z*_*T*_|*x*) and the standard normal distribution. *L*_0_ = −log *p*(*x*|*z*_0_) is the reconstruction loss, which quantifies how well the model can reconstruct the original data. The diffusion loss *L*_*t*_ is given by
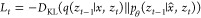
9
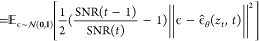
10In practice, however, DDPM training^58^ uses the mean squared error between the true noise and the predicted noise as a simplified training objective with random sampling time step *t* ∼ Uniform(0, *T*):
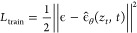
11The predicted noise ϵ̂ consists of two components: ϵ̂^(*x*)^ and ϵ̂^(*h*)^, corresponding to the noise in *x* and *h*, respectively. It can be represented as .

### E(3)-Equivariant Graph Neural Network

The neural network ϕ, which employs an E(3)-equivariant graph neural networks (EGNNs), predicts the noise added from time step *t* – 1 to *t*. The network takes the latent states of the ligand (), protein (), and *interaction particles* (), along with the time step *t*, as input variables in the following way:
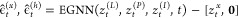
12To preserve rotational and reflectional equivariance, the output must lie on a zero center of gravity subspace. Consequently,  is obtained by subtracting  from the output.^58^

EGNN^56,59^ consists of L layers of equirivariant graph convolutional layers (EGCLs), where ***x***^*l*+1^, ***h***^*l*+1^ = EGCL[***x***^*l*^, ***h***^*l*^]. The coordinates *x* and node type feature *h* at layer *l* are updated as follows:
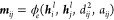
13
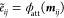
14
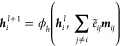
15
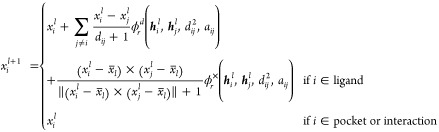
16where ϕ_*e*_, ϕ_att_, ϕ_*h*_ and ϕ_*r*_ are learnable multilayer perceptrons (MLPs), and  and *a*_*ij*_ are the Euclidean distances and edge features between nodes *i* and *j* respectively. Here, EGNN is equivariant with respect to the SE(3) group: EGNN + *b*) = *A* EGNN + *b*, where *A* is an orthogonal rotation matrix and *b* is a translation vector.

### Interaction Particles

**Figure 2:**
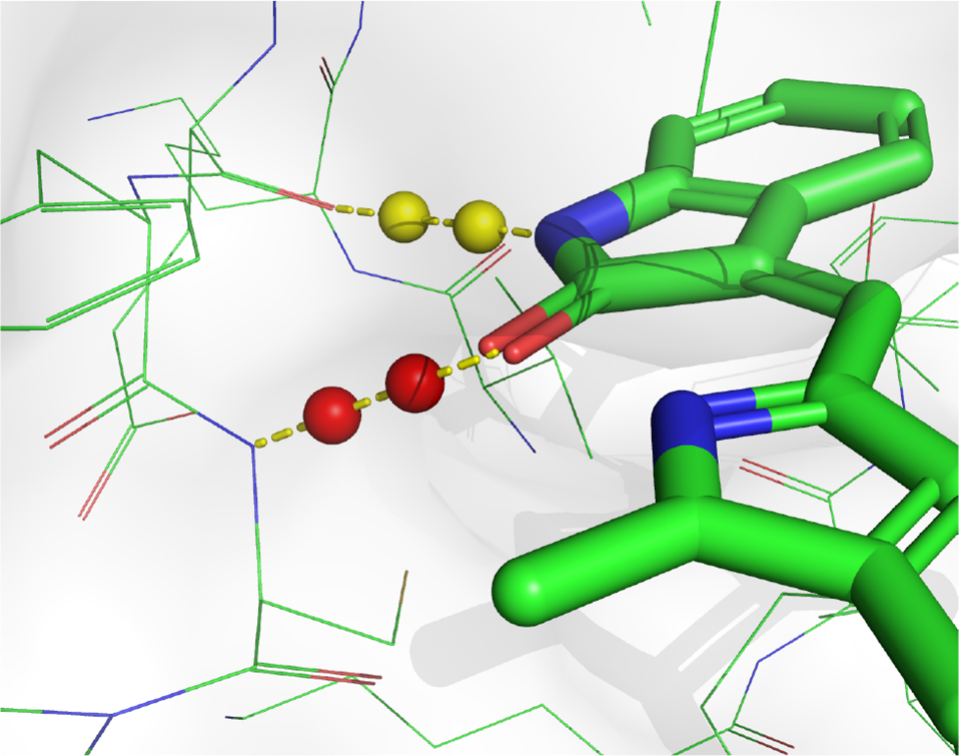
Introduction of *interaction particles* by considering hydrogen bonds as pseudoparticles. Two *interaction particles* are placed on each hydrogen bond (yellow dashed line) between the protein and ligand to provide information about the position and direction of the hydrogen bond. The red and yellow spheres indicate whether the ligand atom is electron-acceptor or electron-donor, respectively.

Hydrogen bonds are introduced into the deep generation model as pseudoparticles called *interaction particles*, with the aim of explicitly incorporating the interactions between proteins and ligand molecules into the diffusion model as shown in Figure 2. When considering hydrogen bonds between protein and ligand molecules,  and  represent the atoms that form the bond belonging to protein and ligand molecules, respectively. The position of *interaction particles* are introduced at positions that divide the distance between  and  into three equal parts:
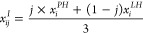
17where ,  and  are the 3D coordinates of ,  and , respectively and *j* denotes the number of *interaction particles* per bond, *j* = 1, 2.

In order to generate ligand molecules to reproduce hydrogen bonds, appropriate ligand atoms  must be placed in the vicinity of a particular protein atoms . If only one *interaction particle* is placed per bond, it can only provide information about the vicinity of the *interaction particle*, whereas by placing two *interaction particles* per bond, it becomes possible to condition the placement of ligand atoms at specific distances and directions from .

In addition, similar to the types of protein and ligand atoms used as node information , *interaction particles* carry node information , whether  is electron-withdrawing or electron-donating
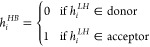
18Thus, it is possible to incorporate *interaction particles* as pseudoparticles into the conditions of the molecule generation model.

The node propagation of *interaction particles* to the ligand molecule by EGNN network makes it possible to propagate the information on the hydrogen bond to the ligand molecule. Through this propagation mechanism, the model learns the relative spatial relationships between *interaction particles*, hydrogen bonding atoms, and their neighboring atoms, which is expected to maintain proper hydrogen bonds in terms of both position and angle.

In the case where only the target protein information is available, without protein–ligand pair data, i.e., no hydrogen bond data, users can place *interaction particles* where they want to place the hydrogen bonds based on the protein structure, and then generate molecules that reproduce the bonds. It is also possible to prevent any existing hydrogen bonds that users do not wish to keep by eliminating the *interaction particles*.

### Data Construction

The CrossDocked data set^69^ contains 22.5 million protein-molecule complex structures. Following by data preparation,^53,70^ the data points with binding pose RMSDs greater than 1 Å are excluded, resulting in a subset of 184,057 data points. Furthermore, the original protein structures are extracted to include only the region within 10 Å around the binding ligand. Then, the protein sequences in this subset are clustered at 30% identity via mmseqs2.^71^ Following the splitting strategies,^70^ 100,000 protein–ligand pairs are randomly selected for the training set, and 100 proteins from the remaining clusters are randomly chosen for the test set. The Open Drug Discovery Toolkit (ODDT)^72^ is applied to the protein–ligand pair data to identify hydrogen bonds. ODDT automatically determines the appropriate bonding directions by examining atomic bonding patterns and hybridization states, without requiring explicit hydrogen atom placements. The *interaction particles* are subsequently determined via the above-mentioned method, with the distributions of hydrogen bonds shown in Figure S1. When identifying hydrogen bonds in the training data and the generated molecule, the target protein uses the full atom representation. When training and inferring the model in this study, however, the target protein is not represented as a full atom, but as a *C*α, which exists one for each residue.

### Training Setting

Our model was trained on 4 GPUs (V100) with the following hyperparameters: the number of diffusion steps (T), batch size, learning rate, number of EGCL layers, hidden state dimensions, and edge cutoff distance are set to 500, 64, 1.0 × 10^–3^, 6, 256, and 5.0 Å, respectively. These hyperparameters are selected on the basis of DiffSBDD values.^59^

### Baselines

Our model is compared with three recent structure-based methods that utilize deep learning methods: Pocket2Mol,^53^ FLAG,^54^ and DiffSBDD.^59^ All these models are GNN-based models that generate 3D molecules. Pocket2Mol and FLAG are autoregressive models that predict the next node on the basis of the protein pocket and the previously placed nodes of the ligand molecule. The key difference between them lies in their ligand molecule representation. Pocket2Mol is an atom-based model, meaning that it generates molecules atom-by-atom, while FLAG is a fragment-based model that constructs molecules by adding valid and realistic fragments sequentially. On the other hand, DiffSBDD is a diffusion model that generates ligand molecules conditioned on the protein pocket. In this comparison, the full-atom model of the protein is adopted. Unlike autoregressive models, where the generated partial structure from the previous step directly influences the next generation, diffusion models generate molecules by simultaneously denoising the entire ligand molecule. This approach allows the model to consider the global context of the molecule during the generation process.

### Evaluation Metrics

Commonly used metrics in evaluation of molecular generation models are adopted, and additional hydrogen bond-related metrics relevant to the objective of this study are incorporated to evaluate the quality of the molecules generated by the models. (1) **Reconstruction of Hydrogen Bond** represents the ability to reconstruct the hydrogen bonds existing in the reference data, i.e., bonds with the correct protein atoms. The pose of the generated molecules is directly used for evaluating hydrogen bond with the protein pocket, without performing any energy minimization steps. This approach allows for evaluation of the quality of the output of the generative model. The number of hydrogen bonds in the reference data varies from 0 to 29 as shown in Figure S1. When evaluating hydrogen bond reproducibility, reference data without hydrogen bonds are excluded, resulting in an assessment of 93 pockets. In some cases, a single correct protein atom may form multiple hydrogen bonds with the generated molecule, potentially resulting in a reproducibility exceeding 100%. (2) **Hydrogen Binding Energy** represents the hydrogen bond contribution to the total binding energy between target protein and ligand molecules. The target of reconstructing hydrogen bonds in this study is to maintain the binding affinity between protein and ligand molecules. However, the reconstruction of the number of hydrogen bonds alone cannot determine whether the reconstructed bonds make a large or small contribution to binding affinity. Hydrogen bonds, as part of intermolecular interactions, contribute to the reduction of binding energy, and the formation of a strong hydrogen bond makes the binding energetically stable and increases the binding affinity between protein and ligand molecules. Therefore, force field calculation via the docking tool is used to determine the strength of the hydrogen bond energy and is used as one of the Metrics. The total binding energy is estimated via Schrödinger’s Glide in XP mode^73,74^ via the in-place docking method and the contribution of the hydrogen bonds is estimated via the SIEVE-Score.^75^ Although commonly used metrics compare docking scores, including pose searches, they are not suitable for evaluating models designed to generate molecules that form hydrogen bonds with specific protein molecules. Therefore, the hydrogen binding energy between protein and ligand molecules is evaluated via the generated pose as is. (3) **High Affinity** indicates the fraction of generated molecules with stronger hydrogen binding energy than the reference data. (4) **QED**(76) is a quantitative estimation of drug-likeness. (5) **SA(synthetic accessibility)**(77) indicates the difficulty of synthesis, and the score is normalized to a range of 0–1, with higher values indicating easier synthesis. (6) **Log*P***(78) represents the logarithm of the partition coefficient of the solvent between octanol and water. Typically, good drug candidates should have a Log *P* value between −0.4 and 5.6.^79^ (7) **Lipinski** calculates how many rules in the Lipinski’s rule of five molecule are satisfied.^80^ (8) **Diversity** measures how varied the set of generated molecules is. It is calculated as the average pairwise dissimilarity (1-Tanimoto Similarity) between all generated molecules for each pocket.^81^ The Tanimoto Similarity is evaluated using RDKitFP^82^ of the molecules.

In this study, we aim to improve the performance of the metrics related to hydrogen bonding while maintaining the performance of the conventional metrics described above.

## Experimental Results and Discussion

DiffInt is compared to three types of deep learning generative models. For evaluating the performance of DiffInt, 100 ligand molecules are generated for each of the 100 proteins in the test set, and the average values of total generated molecules are summarized in Table 1 with respect to the metrics described above. DiffInt outperforms other state-of-the-art models in terms of the reconstruction of hydrogen bonds, hydrogen binding energy and high affinity, although it falls slightly short of the hydrogen energy of the test set. These values are summarized for each pocket in Tables S2 and S3. With respect to drug potentials (QED, SA, Log *P*, and Lipinski), QED, Log *P*, and Lipinski show comparable results to those of the competing model (Pocket2Mol), except for SA. In addition, the molecules generated by DiffInt show the best diversity compared with those generated by the other baseline models.

**Table 1:** Comparison of the Properties of Molecules Generated via the Four Methods^a^

methods	reconstruction of hydrogen bonds (↑)	hydrogen energy (kcal/mol ↓)	high affinity (↑)	QED (↑)	SA (↑)	Lipinski (↑)	log *P*	diversity (↑)
test set		–1.739 ± 1.79		0.476 ± 0.21	0.728 ± 0.14	4.270 ± 1.16	0.894 ± 2.74	
Pocket2Mol	0.276 ± 0.18	–0.589 ± 0.58	0.143 ± 0.22	**0.564** ± **0.15**	**0.749** ± **0.13**	**4.865** ± **0.41**	1.645 ± 1.91	*0.737 ± 0.16*
FLAG	0.368 ± 0.26	–0.765 ± 0.74	0.217 ± 0.23	0.487 ± 0.19	*0.702 ± 0.17*	4.576 ± 0.82	1.438 ± 2.42	0.701 ± 0.14
DiffSBDD	*0.369 ± 0.18*	*–0.940 ± 0.72*	*0.274 ± 0.25*	0.469 ± 0.21	0.578 ± 0.12	4.527 ± 0.92	1.209 ± 2.04	0.728 ± 0.07
DiffInt	**0.732** ± **0.17**	**–1.380** ± **1.26**	**0.338** ± **0.23**	*0.517 ± 0.21*	0.605 ± 0.11	*4.642 ± 0.75*	1.541 ± 2.32	**0.749** ± **0.03**

a The first and second best performances are highlighted in each column.

The addition of the *interaction particles* extracts the desirable structures with respect to the hydrogen binding sites while maintaining molecular diversity. Pocket2Mol and FLAG tend to generate molecules with widely varying diversity bias depending on the pocket, whereas those generated by the diffusion models DiffSBDD and DiffInt produce molecules with relatively high diversity values with small variance as shown in Figure S3. This could be a potential tendency of each deep learning model. The autoregressive models Pocket2Mol and FLAG generate molecules sequentially, and the next node to be added is strongly influenced by the substructures of the molecules generated in the previous steps, especially those chosen in the early steps of the generation process. As a result, molecular generation tends to be biased toward a particular structure for each pocket. In the case of molecular generation for the test set, as shown in Figure S3, the fraction of bias toward a particular structure is much different for each pocket. In other words, the molecules generated in some pockets are very similar, while in others they are not. In contrast, the diffusion models DiffSBDD and DiffInt generate all atoms in parallel, so there is no bias toward the initial structure, as described above, and no diverse variation occurs from pocket to pocket, enabling stable molecule generation.

**Figure 3:**
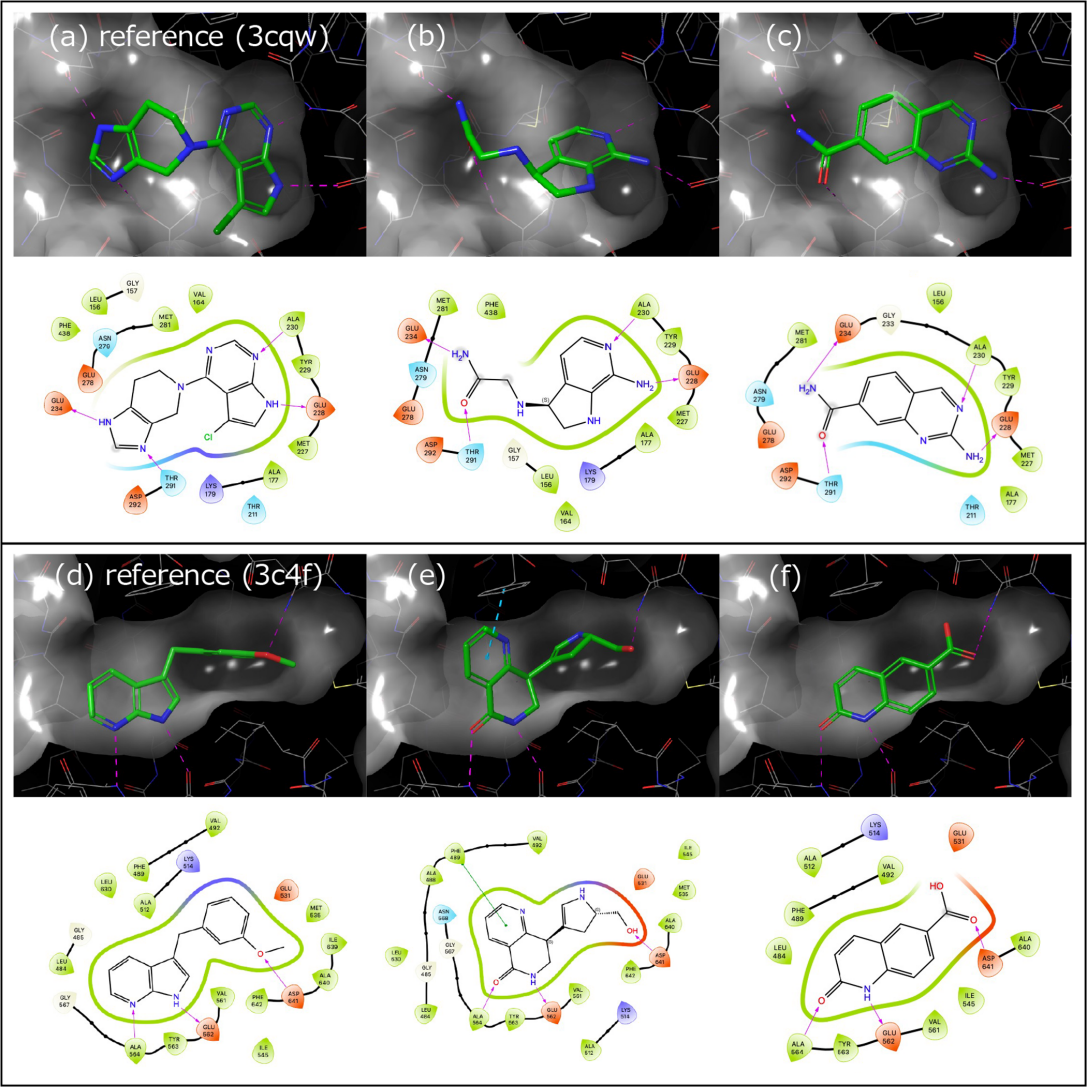
Visualization of ligands with the target proteins (PDB IDs: 3cqw and 3c4f). (a,d) The reference ligand molecules binding to the proteins 3cqw and 4c4f, respectively. (b,c) Example ligands generated by the DiffInt for 3cqw. (e,f) Example ligands generated by the DiffInt for 3c4f. Upper panel in each figure shows 3D structures of the protein–ligand complexes and lower panel illustrates protein–ligand interaction.

Examples of ligand molecule design by DiffInt targeting the drug discovery targets AKT serine/threonine kinase 1 (AKT1, PDB ID: 3CQW) and Fibroblast growth factor receptor 1 (FGFR-1, PDB ID: 3C4F) are shown in Figure 3. The reference ligand of AKT1 forms 4 hydrogen bonds with the target protein: 2 as donors (with GLU234 and GLU228) and 2 as acceptors (with THR291 and ALA230) and generated molecules also have hydrogen bonds to the same residues. The reference ligand of FGFR-1 forms 3 hydrogen bonds with the target protein: 1 as a donor (with GLU562) and 2 as acceptors (with ALA564 and ASP641) and generated molecules also have hydrogen bonds to the same residues.

### Reconstruction of Hydrogen Bonds

It is necessary to place donor and acceptor atoms at appropriate positions and angles for reproducing hydrogen bond between proteins and ligands. By learning the relative positions of *interaction particles*, hydrogen bonding atoms and neighboring atoms through node propagation by EGNN network, the model can reproduce not only the explicitly handled positional information but also the angular information necessary for hydrogen bonding. As a result, DiffInt achieves a significantly higher hydrogen bond reconstruction rate (73.2%) compared to other models as listed in the Table 1.

**Figure 4:**
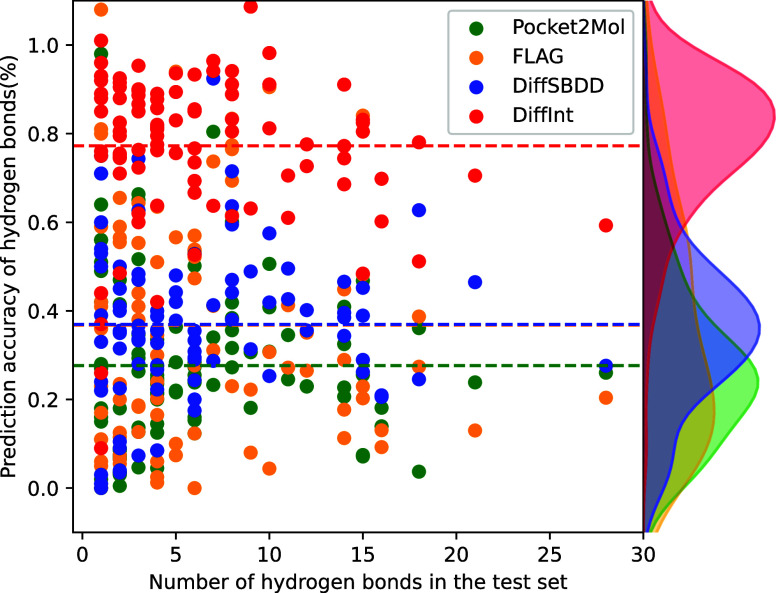
Prediction accuracy of hydrogen bond reproducibility for the four models. Each data point represents the average of hydrogen bond reproducibility for 100 molecules generated per pocket.

The average reconstruction of hydrogen bonds for each pocket is shown in Figure 4, and DiffInt achieves significantly higher prediction accuracy compared to other baseline models across the entire range of hydrogen bond number in the test set ().

Zhao et al.^83^ have reported that the data for virus-suppressing drug molecules extracted from PubChem data set^19^ tends to have six or less intermolecular hydrogen bonds. DiffInt shows remarkably high accuracy of hydrogen bonds in this region as well, achieving an average of 77.2%, which is significantly outperforming the baseline model averages of Pocket2Mol (26.2%), FLAG (36.0%), and DiffSBDD (34.0%).

These results indicate that the baseline models conditioned only on the protein pocket information are insufficient to reconstruct hydrogen bonds. In addition to the protein pocket information, by introducing *interaction particles* in the appropriate form and successfully incorporating hydrogen bonds explicitly into the model, DiffInt has significantly improved its ability to preserve hydrogen bonds.

### Hydrogen Binding Energy

**Figure 5:**
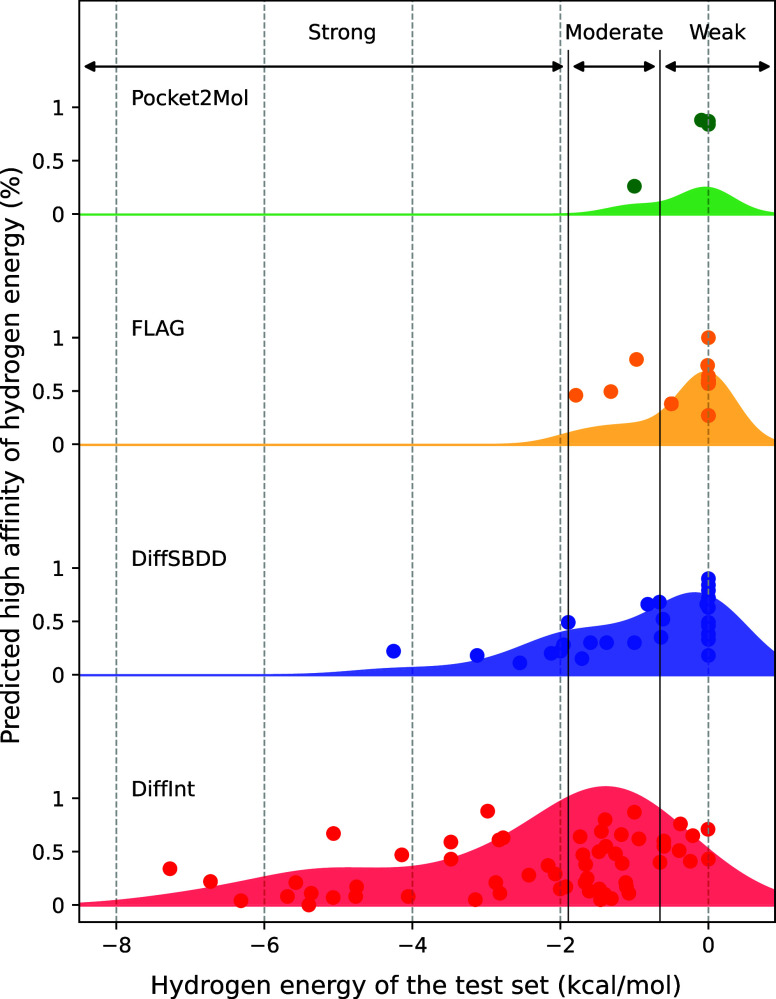
Energy distribution of the best model of high affinity, displayed for each of the four models. Proportion of best models: Pocket2Mol: 4%, FLAG: 12%, DiffSBDD: 27%, DiffInt: 57%.

For a more detailed analysis, the hydrogen binding energies for each individual protein are evaluated. The model with the highest affinity for each pocket is defined as the best model for the pocket, and the distribution of the best model divided into each generation model is shown in Figure 5. The best model for DiffInt covers a wide range of hydrogen binding energies of the test set, significantly higher than those of the other baseline models (DiffSBDD: 27%, FLAG: 12%, Pocket2Mol: 4%) with a result of 57%.

The hydrogen binding energy in the test set varies widely from approximately −8 to 0 kcal/mol, depending on the protein pocket; for example, data points with hydrogen energies near 0 kcal/mol are not significant factors for binding affinity, whereas those with lower hydrogen energies are considered to have higher contributions. DiffInt becomes the best model, especially in regions with low hydrogen energies, and several results with high affinities exceeding 0.5 are observed. On the other hand, other baseline models are concentrated in regions where the hydrogen energies are weak, especially at approximately 0 kcal/mol, resulting in limited contribution to the binding affinity.

**Figure 6:**
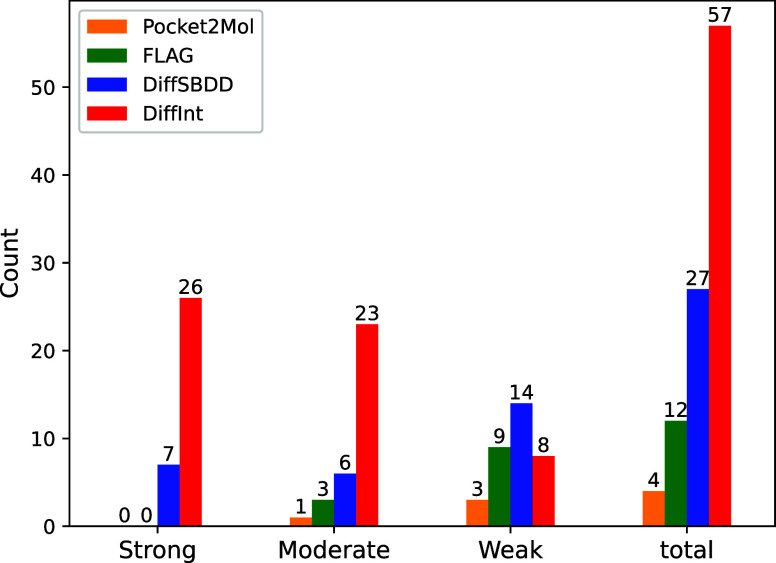
Number of data points becoming the best model for each model across strong, moderate, weak, and total regions.

To clarify the distribution trend of each model, the hydrogen energy in the test set is divided into three equal parts on the basis of the number of entries, labeled the strong, moderate, and weak regions from lowest to highest as shown in Text S2. DiffInt has increasingly larger values as it approaches the strong region, whereas the other base models has the opposite trend, as shown in Figure 6.

DiffInt outperforms the other baseline models in the strong and moderate regions, while other baseline models show better performance in the weak region. This difference stems from DiffInt’s explicit modeling of interactions, which effectively captures hydrogen bonding features that contribute to binding affinity in stronger regions.

### Substructure Analysis

**Figure 7:**
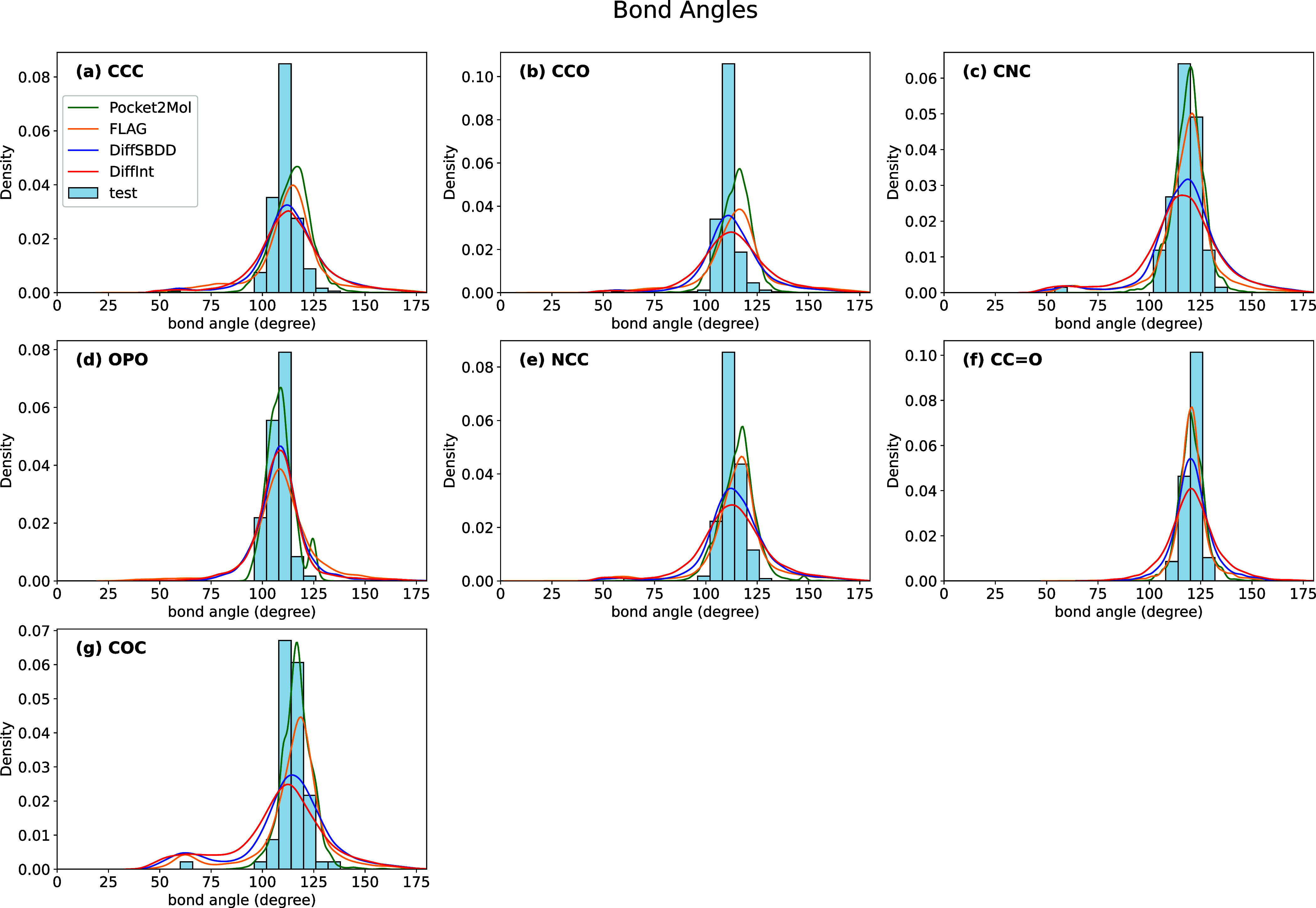
Bond angle distributions for generated molecules and those in the test set. Density plots of the bond angles for (a) CCC, (b) CCO, (c) CNC, (d) OPO, (e) NCC, (f) CC=O, and (g) COC. Distributions are shown for molecules generated by four models (Pocket2Mol, FLAG, DiffSBDD, and DiffInt) and compared to the test set.

**Figure 8:**
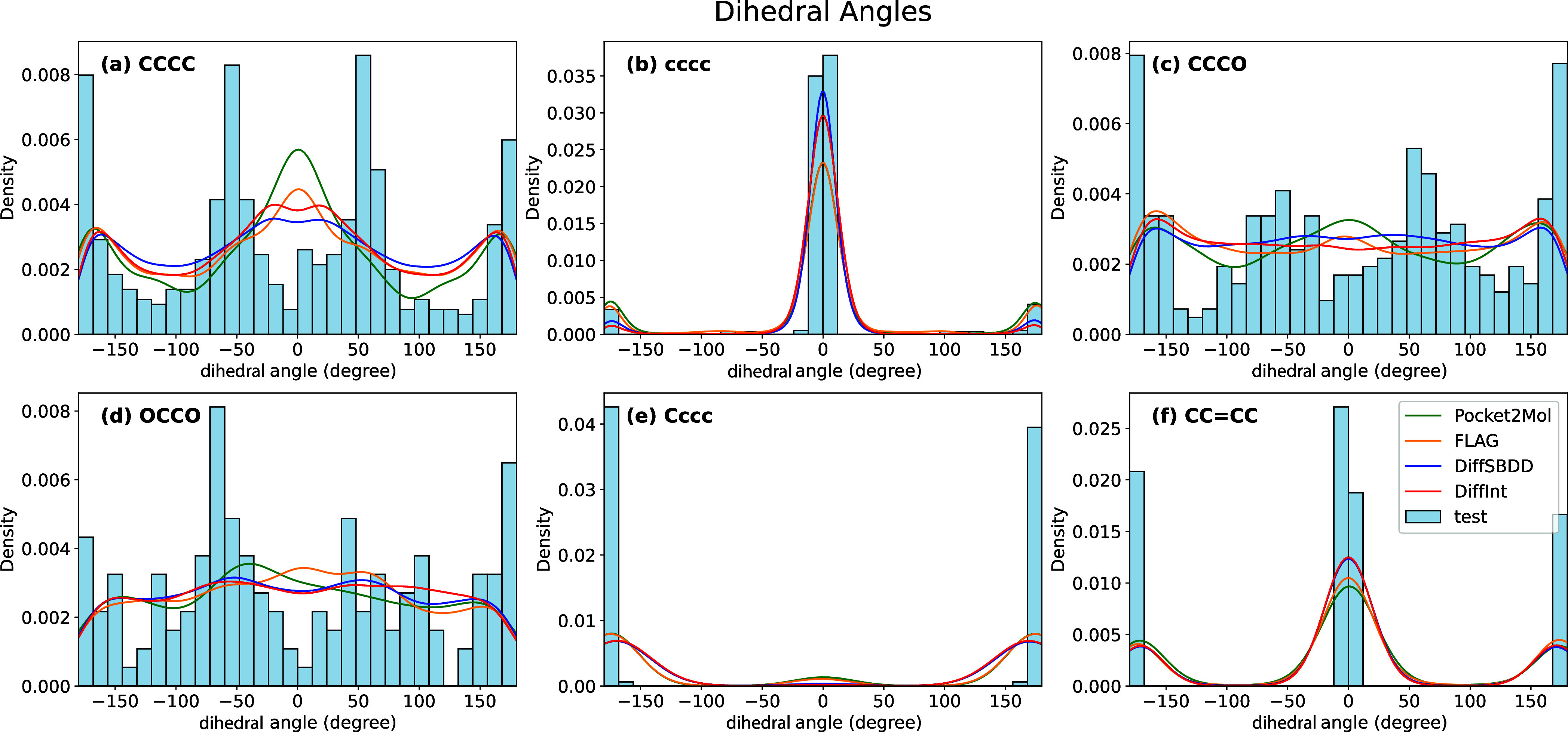
Dihedral angle distributions for generated molecules and those in the test set. Density plots of the dihedral angles for (a) CCCC, (b) cccc, (c) CCCO, (d) OCCO, (e) Cccc, and (f) CC=CC. Distributions are shown for molecules generated by four models (Pocket2Mol, FLAG, DiffSBDD, and DiffInt) and compared to the test set. The lower letters represent the atoms in the aromatic rings.

Molecules generated by generative models often exhibit instability and unrealistic structures. To evaluate the 3D stability of generated molecules, the distributions of bond angles and dihedral angles are compared between the generated molecules and those in the test set. Specified bond pairs and bond triples within molecules are identified, and bond angles and dihedral angles are calculated using RDKit.^82^ The bond angles for seven types (CCC, CCO, CNC, OPO, NCC, CC=O, COC) are shown in Figure 7, and the dihedral angles for six types (CCCC, cccc, CCCO, OCCO, Cccc, CC=CC) are shown in Figure 8. Among the four generating models, Pocket2Mol shows a similar distribution to the test set with respect to the bond angles, while all models show similar performance for the dihedral angles. However, across the entire range of bond and dihedral angles, the molecules produced by generative models do not fully reproduce the results of the test set. Comparing the two diffusion models, DiffSBDD and DiffInt, the performance is nearly equivalent performance, indicating that the incorporation of *interaction particles* into the model does not significantly change the performance of 3D stability.

### Atom-Type Analysis

The formation of the hydrogen bonds between protein and ligand molecules require the appropriate donor and acceptor atoms to be placed at the appropriate position and angle on the ligand molecules with respect to the target protein. It might be assumed that an excess of these atoms could enhance hydrogen bonding. The one of the key indicators of drug-likeness, Lipinski’s Rule of Five,^80^ suggests that drug candidates typically have less than five donor atoms and less than ten acceptor atoms. Therefore, the generation of ligand molecules should avoid the excess of these atoms. The fractions of donor and acceptor atoms in the training set, test set, and generated molecules are listed in Table 2. DiffInt, which introduces *interaction particles*, shows similar donor and acceptor atom ratios to the training and test sets, and does not contain more of these atoms than the other generative models. Thus, DiffInt has no bias toward excessive placement of those atoms in order to obtain hydrogen bonds. Considering this result in conjunction with the hydrogen bond reproducibility mentioned above, it suggests that DiffInt is a model that places the right atoms in the right positions for hydrogen bonds with the target protein.

**Table 2:** Fractions of Donor and Acceptor Atoms

	donor	acceptor
training	0.128	0.236
test	0.158	0.269
Pocket2Mol	0.109	0.220
FLAG	0.138	0.233
DiffSBDD	0.131	0.248
DiffInt	0.137	0.223

## Conclusions

In this work, we propose DiffInt, preserving hydrogen bonds between protein pockets and ligand molecules with an E(3)-conditional diffusion model. By introducing pseudoparticles called *interaction particles*, we have successfully incorporated hydrogen bond information explicitly into the deep generation model. DiffInt demonstrates the ability to significantly outperform the baseline models for hydrogen bond reconstruction and high binding affinity. DiffInt also has an advantage in generating molecules with strong hydrogen binding energies, which is expected to contribute highly to the protein binding affinity crucial for drug discovery. The trained models in this study are publicly available so researchers can use the models without retraining, however if they need to retrain user-specific models on their own data, they will need large-scale GPUs. This method is not applicable to those whose binding site is unknown, so it is necessary to determine it with other methods. This study deals only with hydrogen bonding, and other interactions will be incorporated into the model using approaches analogous to those developed in DiffInt. In summary, DiffInt can generate a wide variety of molecules while maintaining high hydrogen bond reproducibility, suggesting that it has the potential to be a useful tool for structure-based drug design.

## Data Availability

To facilitate the use of our tool for generating new drug molecules based on any protein three-dimensional structure, we have made the source code and trained model available on GitHub (https://github.com/sekijima-lab/DiffInt) under the MIT license, with the execution environment provided on Google Colab.

## Supporting Information Available

Distributions of the number of the hydrogen bonds; definition of the strong, moderate and weak regions of the hydrogen binding energy of the test set; distribution of the hydrogen binding energy of the test set; differences in the diversity of generated molecules across models; average distributions of generated molecule diversity per pocket across the four models; number of the hydrogen bonds for each protein pocket; and hydrogen binding energy and high affinity for each protein pocket (PDF)
