# Supplementary material for: DiffInt: A Diffusion Model for Structure-Based Drug Design with Explicit Hydrogen Bond Interaction Guidance
Source: J Chem Inf Model. 2024 Dec 19;65(1):71–82. doi: 10.1021/acs.jcim.4c01385 (PMC11733934; doi:10.1021/acs.jcim.4c01385)
Supplement: Supplementary file 1 — ci4c01385_si_001.pdf [file ci4c01385_si_001.pdf]

Supporting information: DiffInt: A Diffusion Model for Structure-Based Drug Design with Explicit Hydrogen Bond Interaction Guidance

Masami Sako,† Nobuaki Yasuo,‡ and Masakazu Sekijima*,†

†*Department of Computer Science, Institute of Science Tokyo, Yokohama, Kanagawa, Japan*

‡*Academy for Convergence of Materials and Informatics (TAC-MI), Institute of Science Tokyo, Meguro-ku, Tokyo, Japan*

E-mail: sekijima@comp.isct.ac.jp

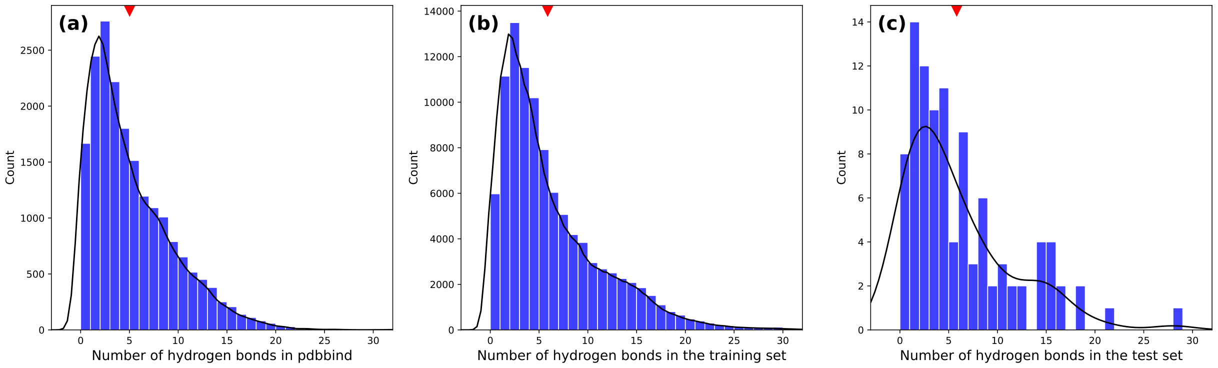

Figure S1: Distribution of hydrogen bonds across three types of dataset. (a) PDBBind dataset: Among 19443 data, 1667 (8.6 %) contain zero hydrogen bond. (b) training set: Among 99918 data, 5976 (6.0 %) contain zero hydrogen bond. (c) test set: Among 100 data, 8 (8.0 %) contain zero hydrogen bond. Red triangles indicate the mean value.

Text S2. Distribution of hydrogen bonding energy

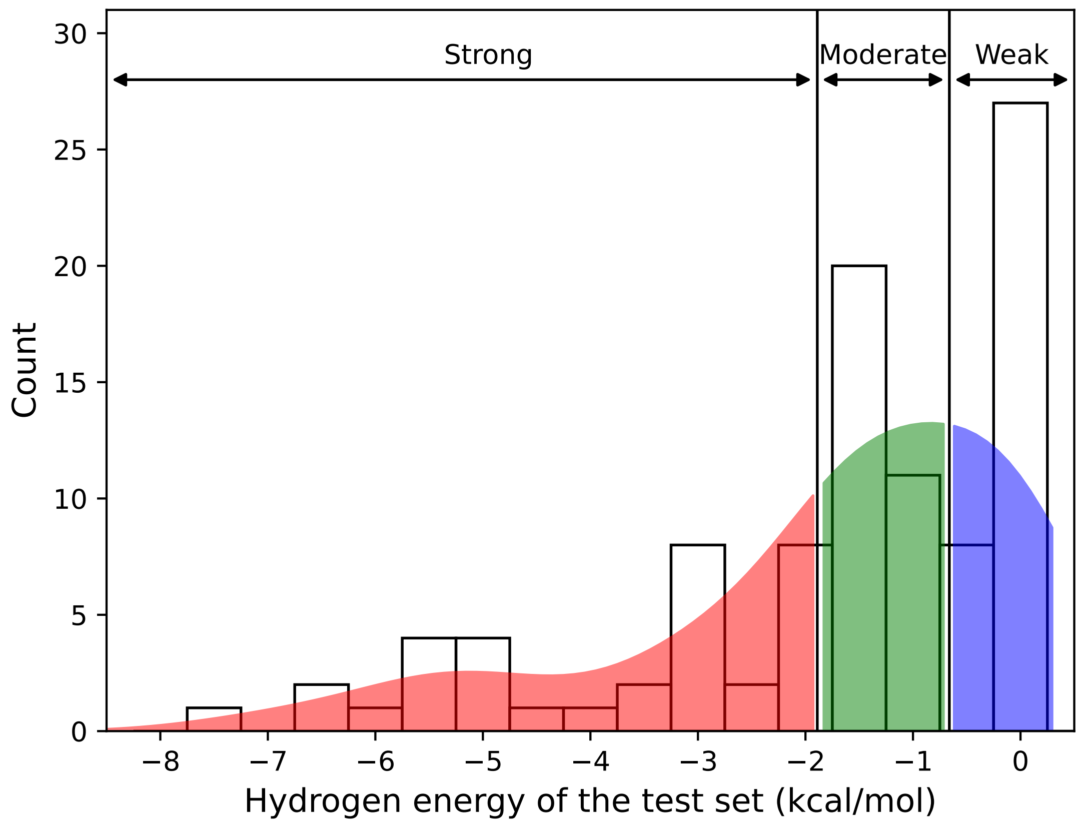

Figure S2: Hydrogen binding energy for the test set. To divide the data points in the test set into three equal parts, the lowest value region is defined as 'strong', the medium as 'moderate', and the highest as 'weak'.

The distribution of the hydrogen binding energy for the test set is shown in Figure S2. The distribution extends from -7.28 to 0 kcal/com, with 70 % of the data concentrated in the -2 to 0 kcal/mol region. The hydrogen binding energy is the sum of the hydrogen bonds between the ligand and protein. The larger the absolute value of the hydrogen energy is, the higher the contribution to the binding affinity and the greater difficulty of reproduction by the generated molecules. Like the number of hydrogen bonds, to assess the model performance by the difference in the magnitude of the hydrogen binding energy, the hydrogen binding energy is divided into three equal regions so that the number of data points in each region

is equal. The regions are defined as follows: from -8 to -2.1 kcal/mol as "strong", from -2.1 to -1.0 kcal/mol as "moderate", and from -1.0 to 0 kcal/mol as "weak".

Text S3. Diversity of generated molecules

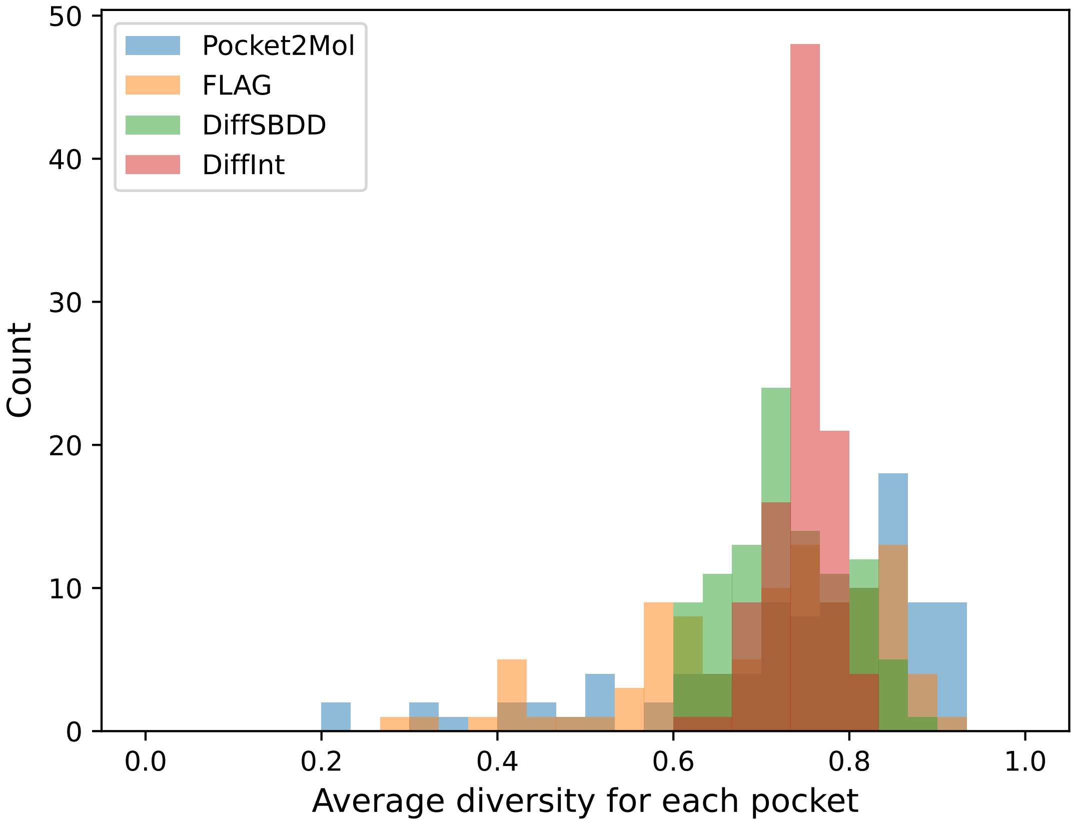

Figure S3: Average distribution of generated molecule diversity per pocket across the four models.

The average diversity for generated molecules for each pocket is shown in Figure S3. Pocket2Mol[1] and FLAG[2] show a wide distribution of diversity, whereas DiffSBDD[3] and DiffInt show narrow distributions with high diversity. The former models are strongly influenced by substructures of molecules generated in the previous steps due to sequential generation and sometimes generate similar molecules. As a result, they may have low diversity for certain pockets. The latter models generate all atoms of molecules in parallel, so there is no bias, as described above; thus, the generated molecules show high diversity on average.

Table S1: The Number of the hydrogen bonds for each protein pocket. The results for the four models show the average value of generated molecules

PDB	Number of Hydrogen bonds				
IDs	test set	Pockt2Mol	FLAG	DiffSBDD	DiffInt
14gs	2	0.36	0.13	0.45	1.59
1a2g	1	0.64	0.23	0.71	1.01
1afs	1	0.42	0.81	0.75	0.93
1ai4	6	1.64	0.00	1.77	4.00
1coy	0	-	-	-	-
1d7j	6	0.96	0.74	1.74	4.16
1djy	12	2.76	4.21	4.82	9.31
1dxo	2	0.14	0.23	0.07	1.51
1e8h	11	3.80	2.99	5.45	7.76
1fmc	4	0.50	2.04	1.07	3.51
1gg5	1	0.56	1.08	0.60	0.93
1h0i	3	0.91	0.56	2.23	2.60
1h36	0	-	-	-	-
1jn2	8	4.81	6.67	5.72	7.29
1k9t	0	-	-	-	-
1l3l	2	0.07	0.11	0.63	1.42
1phk	16	2.23	1.48	3.29	11.17
1r1h	14	3.18	4.05	5.54	9.60
1rs9	4	0.94	1.20	1.42	3.10
1umd	15	1.12	12.61	5.84	12.07
2azy	2	0.01	0.40	0.08	1.61
2cy0	14	5.73	2.48	6.52	12.75
2e24	0	-	-	-	-
2e6d	7	5.63	2.88	6.47	6.75

PDB	Number of Hydrogen bonds				
IDs	test set	Pockt2Mol	FLAG	DiffSBDD	DiffInt
2f2c	6	1.43	3.22	1.20	4.41
2gns	1	0.15	0.00	0.01	0.26
2hcj	21	5.01	2.73	9.76	14.81
2jjg	8	3.35	6.12	5.09	6.66
2pc8	8	3.07	5.55	3.76	7.53
2pqw	3	0.14	1.23	0.22	2.29
2rhy	1	0.18	0.80	0.22	0.89
2rma	4	0.97	0.24	1.10	3.05
2v3r	3	0.79	1.66	1.88	2.55
2z3h	16	2.90	2.09	3.35	9.63
2zen	1	0.16	0.06	0.53	0.85
3af2	15	7.02	7.23	6.78	12.37
3b6h	0	-	-	-	-
3chc	6	1.46	3.13	1.46	5.13
3daf	11	2.70	4.54	4.69	6.71
3dzh	9	1.63	0.72	2.82	5.68
3ej8	3	1.55	1.32	1.05	2.69
3g51	15	3.83	3.04	3.93	7.26
3gs6	10	4.08	9.05	5.75	9.82
3hy9	8	2.18	4.93	3.04	6.44
3jyh	3	1.04	0.38	1.41	2.70
3kc1	10	5.06	0.44	4.19	8.12
3l3n	8	2.85	6.19	4.76	7.11
3li4	1	0.98	0.59	0.71	0.76
3nfb	9	2.76	2.00	4.40	9.78
3o96	4	0.18	0.05	0.34	1.68
3pdh	3	0.41	1.66	1.10	2.17

PDB	Number of Hydrogen bonds				
IDs	test set	Pockt2Mol	FLAG	DiffSBDD	DiffInt
3pnm	5	1.82	0.50	1.89	4.47
3tym	4	0.80	1.20	0.34	2.55
3u5y	6	1.53	3.23	1.85	4.60
3u9f	1	0.28	0.36	0.54	0.96
3v4t	14	4.55	1.58	5.39	10.42
3w83	3	1.95	2.70	1.45	2.66
4aaw	6	3.01	2.84	3.17	5.60
4aua	2	0.83	1.31	0.80	0.97
4azf	3	0.38	1.14	0.84	1.80
4bel	7	2.38	2.18	2.89	6.59
4d7o	2	0.13	0.47	0.18	1.83
4f1m	6	0.92	1.66	1.05	3.16
4g3d	6	1.70	3.20	2.13	5.11
4gvd	0	-	-	-	-
4h3c	1	0.00	0.11	0.03	0.75
4iiy	15	1.07	3.44	4.34	12.47
4iwq	4	0.85	0.96	0.89	3.24
4ja8	4	0.58	0.10	1.11	3.26
4kcq	1	0.56	0.17	0.24	0.92
4keu	5	1.42	0.37	2.40	4.15
4lfu	2	0.06	1.49	0.70	1.65
4m7t	12	2.75	3.18	4.27	8.72
4p6p	18	6.50	6.97	11.29	14.05
4pxz	14	2.89	6.28	4.81	10.81
4q8b	0	-	-	-	-
4qlk	18	0.67	4.93	4.42	9.21
4rlu	4	0.86	0.66	1.55	3.18

PDB	Number of Hydrogen bonds				
IDs	test set	Pockt2Mol	FLAG	DiffSBDD	DiffInt
4rn0	1	0.02	0.41	0.00	0.09
4rv4	28	7.29	5.71	7.73	16.60
4tos	2	0.15	0.15	0.82	1.81
4tqr	3	0.88	0.55	1.01	2.86
4u5s	1	0.49	0.11	0.39	0.88
4xli	3	1.99	1.93	1.00	1.86
4yhj	4	0.99	1.38	1.31	3.28
4z2g	4	1.34	0.81	1.43	3.56
4zfa	1	0.27	0.05	0.33	0.37
5aeh	2	0.18	1.18	0.90	1.76
5b08	4	1.02	2.54	1.60	3.43
5bur	10	3.08	3.07	2.53	9.11
5d7n	5	1.08	2.83	2.21	3.78
5i0b	1	0.51	0.42	0.50	0.44
5l1v	5	1.10	4.70	2.10	4.68
5liu	2	0.17	1.11	0.71	1.49
5mgl	2	0.94	1.13	1.00	1.85
5mma	0	-	-	-	-
5ngz	0	-	-	-	-
5q0k	2	0.07	0.25	0.21	1.50
5tjn	7	1.63	5.16	2.01	4.46
5w2g	8	2.53	1.84	3.53	4.91

Table S2: The hydrogen binding energy and high affinity for each protein pocket. The results for the four models show the average value of generated molecules

PDB	Hydrogen Energy (kcal/mol)					High Affinity			
IDs	test set	Pockt2Mol	FLAG	DiffSBDD	DiffInt	Pockt2Mol	FLAG	DiffSBDD	DiffInt
14gs	-0.378	-0.115	-0.266	-0.420	-0.568	0.17	0.230	0.44	0.760
1a2g	0.000	-0.060	-0.124	-0.446	-0.110	0.20	0.570	0.56	0.270
1afs	-0.603	-0.149	-0.526	-0.445	-0.731	0.06	0.463	0.38	0.600
1ai4	-1.401	-0.419	-0.071	-0.317	-1.205	0.06	0.010	0.05	0.100
1coy	0.000	-0.090	-0.084	-0.688	-0.091	0.29	0.180	0.72	0.220
1d7j	-1.479	-0.221	-0.088	-0.644	-1.463	0.01	0.000	0.10	0.500
1djy	-1.659	-0.445	-0.484	-0.991	-1.568	0.00	0.020	0.22	0.380
1dxo	-0.211	-0.093	-0.030	-0.141	-0.377	0.18	0.042	0.20	0.650
1e8h	-5.576	-2.138	-1.682	-2.756	-4.398	0.00	0.000	0.05	0.210
1fmc	-1.447	-0.033	-0.851	-0.516	-1.538	0.00	0.141	0.09	0.690
1gg5	-0.602	-0.129	-0.530	-0.413	-0.608	0.03	0.442	0.36	0.550
1h0i	-0.662	-0.498	-0.346	-1.071	-0.815	0.37	0.170	0.68	0.640
1h36	0.000	-0.094	-0.085	-0.198	-0.042	0.21	0.276	0.33	0.100
1jn2	-1.894	-1.551	-1.914	-1.937	-1.712	0.39	0.470	0.49	0.320
1k9t	0.000	-0.107	-0.250	-0.428	-0.199	0.34	0.350	0.72	0.500
1l3l	-1.458	-0.544	-0.024	-0.387	-0.446	0.00	0.000	0.04	0.050
1phk	-2.548	-1.049	-0.930	-1.483	-1.554	0.03	0.000	0.11	0.080
1r1h	-2.426	-0.743	-0.757	-1.132	-1.847	0.00	0.032	0.06	0.280
1rs9	-0.396	-0.186	-0.290	-0.385	-0.434	0.18	0.390	0.36	0.510
1umd	-5.688	-0.715	-4.346	-2.825	-3.664	0.00	0.042	0.05	0.080
2azy	-0.245	-0.083	-0.081	-0.052	-0.325	0.11	0.140	0.07	0.410
2cy0	-2.873	-1.132	-0.625	-1.522	-2.423	0.01	0.000	0.09	0.210
2e24	-1.734	-0.182	-0.981	-0.848	-1.876	0.00	0.071	0.10	0.640
2e6d	-5.066	-3.368	-1.582	-3.648	-4.879	0.08	0.000	0.12	0.670

PDB	Hydrogen Energy (kcal/mol)					High Affinity			
IDs	test set	Pockt2Mol	FLAG	DiffSBDD	DiffInt	Pockt2Mol	FLAG	DiffSBDD	DiffInt
2f2c	-1.611	-0.691	-1.168	-0.805	-1.109	0.04	0.109	0.11	0.130
2gns	0.000	-0.012	-0.182	-0.177	-0.122	0.07	0.420	0.28	0.430
2hcj	-7.276	-1.801	-1.091	-3.418	-5.515	0.00	0.000	0.01	0.340
2jjg	-1.256	-0.576	-1.083	-1.168	-1.257	0.09	0.320	0.43	0.480
2pc8	-2.983	-1.847	-2.831	-2.082	-3.652	0.10	0.485	0.24	0.880
2pqw	-0.006	-0.052	-0.123	-0.369	-0.340	0.13	0.465	0.46	0.710
2rhy	0.000	-0.013	-0.067	-0.266	-0.167	0.04	0.242	0.49	0.440
2rma	-1.958	-0.194	-0.341	-1.237	-0.718	0.00	0.010	0.28	0.070
2v3r	0.000	-0.256	-0.721	-1.149	-0.394	0.58	1.000	0.93	0.650
2z3h	-5.400	-1.075	-0.389	-1.409	-3.199	0.00	0.000	0.00	0.000
2zen	0.000	-0.420	-0.455	-0.808	-0.140	0.69	0.657	0.90	0.420
3af2	-4.851	-2.315	-2.576	-2.176	-4.380	0.10	0.020	0.03	0.470
3b6h	0.000	-0.048	-0.184	-0.395	-0.114	0.18	0.350	0.46	0.240
3chc	-0.654	-0.345	-0.250	-0.343	-0.601	0.23	0.121	0.22	0.400
3daf	-3.034	-0.297	-0.891	-1.442	-2.055	0.00	0.020	0.20	0.060
3dzh	-3.126	-0.574	-0.480	-1.975	-1.317	0.00	0.000	0.18	0.010
3ej8	-2.070	-0.896	-0.637	-0.431	-1.688	0.05	0.020	0.02	0.290
3g51	-5.842	-1.438	-1.316	-1.646	-2.719	0.00	0.000	0.00	0.070
3gs6	-1.396	-0.528	-1.222	-1.072	-1.603	0.02	0.354	0.32	0.800
3hy9	-3.149	-0.570	-1.311	-1.041	-1.735	0.00	0.000	0.00	0.050
3jyh	-1.075	-0.323	-0.151	-0.345	-0.434	0.03	0.010	0.10	0.110
3kc1	-3.480	-1.357	-0.072	-1.490	-3.085	0.01	0.000	0.08	0.590
3l3n	-2.830	-0.631	-1.880	-1.717	-2.758	0.00	0.374	0.15	0.610
3li4	0.000	-0.489	-0.240	-0.582	-0.530	0.87	0.680	0.82	0.750
3nfb	-4.057	-1.278	-0.556	-1.225	-3.038	0.00	0.000	0.02	0.080
3o96	0.000	-0.256	-0.345	-0.535	-0.204	0.84	0.450	0.72	0.400
3pdh	-1.500	-0.164	-1.264	-0.489	-0.990	0.12	0.380	0.19	0.040

PDB	Hydrogen Energy (kcal/mol)					High Affinity			
IDs	test set	Pockt2Mol	FLAG	DiffSBDD	DiffInt	Pockt2Mol	FLAG	DiffSBDD	DiffInt
3pnm	-1.176	-0.647	-0.381	-0.640	-1.283	0.12	0.050	0.19	0.660
3tym	-1.117	-0.091	-0.545	-0.211	-0.728	0.00	0.200	0.02	0.210
3u5y	-3.479	-0.908	-2.952	-1.488	-3.337	0.00	0.242	0.03	0.430
3u9f	0.000	-0.066	-0.287	-0.194	-0.110	0.14	0.580	0.40	0.260
3v4t	-4.767	-0.834	-0.314	-1.857	-3.116	0.00	0.000	0.01	0.080
3w83	-0.094	-0.892	-0.500	-0.719	-0.447	0.88	0.536	0.75	0.590
4aaw	-1.000	-1.331	-1.044	-1.058	-1.859	0.79	0.410	0.53	0.870
4aua	-1.638	-0.779	-1.133	-0.671	-1.091	0.11	0.050	0.11	0.250
4azf	-1.376	-0.740	-1.280	-0.984	-0.685	0.11	0.300	0.30	0.050
4bel	-2.167	-0.813	-1.021	-1.458	-1.975	0.01	0.020	0.23	0.370
4d7o	-1.108	-0.069	-0.495	-0.241	-0.580	0.02	0.120	0.04	0.170
4f1m	-2.003	-0.909	-1.337	-0.968	-1.405	0.01	0.080	0.10	0.150
4g3d	-0.972	-0.611	-1.336	-1.125	-0.930	0.28	0.796	0.52	0.330
4gvd	0.000	-0.194	-0.298	-0.310	-0.269	0.55	0.600	0.63	0.530
4h3c	0.000	0.000	-0.061	-0.080	-0.048	0.00	0.270	0.22	0.240
4iiy	-2.774	-0.285	-0.584	-1.102	-3.036	0.00	0.020	0.04	0.630
4iwq	-1.927	-0.747	-0.810	-0.756	-1.433	0.03	0.010	0.07	0.170
4ja8	-1.311	-0.021	-0.069	-0.201	-0.669	0.00	0.000	0.00	0.060
4kcq	-0.011	-0.090	-0.880	-0.555	-0.140	0.34	0.740	0.72	0.360
4keu	-0.640	-0.332	-0.178	-0.574	-0.360	0.18	0.120	0.35	0.150
4lfu	0.000	-0.068	-0.179	-0.109	-0.264	0.10	0.602	0.32	0.520
4m7t	-4.755	-0.747	-1.023	-1.495	-3.336	0.00	0.000	0.00	0.170
4p6p	-4.254	-1.761	-2.457	-3.101	-2.936	0.01	0.050	0.22	0.060
4pxz	-5.367	-0.401	-1.426	-1.414	-3.617	0.00	0.010	0.00	0.110
4q8b	0.000	-0.053	-0.001	-0.183	-0.086	0.15	0.023	0.38	0.250
4qlk	-6.317	-0.322	-1.257	-1.388	-3.052	0.00	0.000	0.00	0.040
4rlu	-0.615	-0.131	-0.151	-0.722	-0.681	0.05	0.100	0.52	0.430

PDB	Hydrogen Energy (kcal/mol)					High Affinity			
IDs	test set	Pockt2Mol	FLAG	DiffSBDD	DiffInt	Pockt2Mol	FLAG	DiffSBDD	DiffInt
4rn0	-0.023	-0.351	-0.148	-0.345	-0.171	0.56	0.412	0.66	0.440
4rv4	-6.729	-1.784	-2.278	-1.874	-4.263	0.00	0.000	0.00	0.220
4tos	-2.000	-0.902	-0.429	-1.267	-1.873	0.01	0.000	0.22	0.200
4tqr	-0.940	-0.330	-0.388	-0.661	-0.979	0.08	0.200	0.30	0.620
4u5s	0.000	-0.495	-0.300	-0.565	-0.215	0.70	0.590	0.79	0.510
4xli	-1.697	-0.888	-1.255	-0.716	-1.547	0.03	0.385	0.03	0.470
4yhj	-2.818	-0.686	-1.273	-1.162	-2.191	0.00	0.000	0.03	0.110
4z2g	-0.820	-0.674	-0.291	-1.083	-0.520	0.40	0.120	0.66	0.300
4zfa	0.000	-0.024	-0.397	-0.233	-0.065	0.07	0.600	0.38	0.110
5aeh	-1.387	-0.416	-0.571	-0.804	-1.308	0.01	0.130	0.25	0.550
5b08	-1.318	-0.366	-1.234	-0.691	-1.253	0.03	0.494	0.18	0.430
5bur	-1.709	-0.871	-0.668	-0.868	-1.068	0.07	0.020	0.15	0.100
5d7n	-1.166	-0.230	-0.896	-0.673	-1.094	0.03	0.293	0.19	0.390
5i0b	-1.000	-0.924	-0.482	-0.639	-0.775	0.26	0.180	0.23	0.180
5l1v	-1.791	-0.602	-1.697	-1.188	-1.692	0.00	0.460	0.23	0.394
5liu	-1.474	-0.409	-0.647	-0.648	-1.040	0.01	0.077	0.12	0.150
5mgl	-1.000	-0.715	-0.652	-0.828	-0.958	0.14	0.090	0.30	0.270
5mma	0.000	-0.289	-0.083	-0.670	-0.253	0.43	0.220	0.84	0.530
5ngz	0.000	-0.413	-0.383	-0.405	-0.115	0.59	0.640	0.57	0.250
5q0k	0.000	-0.044	-0.027	-0.078	-0.011	0.08	0.110	0.18	0.060
5tjn	-1.666	-0.310	-0.884	-0.301	-1.287	0.01	0.131	0.01	0.210
5w2g	-1.594	-0.744	-0.737	-1.212	-1.142	0.01	0.070	0.30	0.260

References

(1) Peng, X.; Luo, S.; Guan, J.; Xie, Q.; Peng, J.; Ma, J. Pocket2mol: Efficient molecular sampling based on 3d protein pockets. International Conference on Machine Learning. 2022; pp 17644–17655.

(2) ZHANG, Z.; Min, Y.; Zheng, S.; Liu, Q. Molecule Generation For Target Protein Binding with Structural Motifs. The Eleventh International Conference on Learning Representations. 2023.

(3) Schneuing, A.; Du, Y.; Harris, C.; Jamasb, A.; Igashov, I.; Du, W.; Blundell, T.; Lió, P.; Gomes, C.; Welling, M.; others Structure-based drug design with equivariant diffusion models. *arXiv preprint arXiv:2210.13695* **2022**,
